# Fecundity of a Free-Martin

**Published:** 1852-02

**Authors:** Joseph Moore

**Affiliations:** Jefferson Medical College, and of Cumberland Co., New Jersey; Philadelphia


*Fecundity of a Free-Martin.* By JOSEPH MOORE, of the Jefferson Medical College, and of Cumberland Co., New Jersey. (Communicated by Professor Dunglison.)

Professor Dunglison,

DEAR SIR,—I herewith forward you a newspaper, published in the town where I reside, in which is a communication from a very intelligent farmer, relating to a free-martin which he raised on his farm, and which not only received the male, but gave birth to a healthy offspring.

The gentleman who is the author of the communication, I have known for several years, and can therefore vouch both for his intelligence and veracity.

As a knowledge of such cases is not devoid of interest to persons devoted to scientific pursuits, I write the accompanying note, for the purpose of adding my testimony to the truth of the gentleman's communication, as he is, most likely, unknown to yourself. Very respectfully,

JOSEPH MOORE.

*Philadelphia, Jan. 7th,* 1852.

From the West Jersey Pioneer, Oct. 15th, 1851.

A NUT FOR THE CURIOUS.—On the 5th of the 6th month, 1849, one of my cows dropped two calves, a male and female, and on the 11th inst. the latter dropped a calf, healthy and strong, and has the appearance of making a good cow. I have never heard of a similar instance.

Respectfully, BENJAMIN SHEPPARD.

This case disproves the correctness of the commonly received opinion, that the female of twin calves is invariably barren and unfruitful.

